# Muscle oxygenation measured by near‐infrared spectroscopy during resistance training: A scoping review

**DOI:** 10.14814/phy2.71014

**Published:** 2026-07-14

**Authors:** Moath Mohammad, Ghydaa Alshanty, Juliette Langelier, Morgane Mary‐Pouliot, Michael Stolberg, Rami Hammad, Alain Steve Comtois

**Affiliations:** ^1^ Département des Sciences de l'Activité Physique Université du Québec à Montréal Montréal Canada; ^2^ Département des Sciences Biologiques Université du Québec à Montréal Montréal Canada; ^3^ Department of Physiotherapy, School of Rehabilitation Sciences The University of Jordan Amman Jordan; ^4^ Kinesiology and Training Department, School of Sport Sciences University of Jordan Amman Jordan

**Keywords:** internal load monitoring, muscle oxygenation, NIRS, resistance training

## Abstract

Near‐infrared spectroscopy (NIRS) is a noninvasive optical technique used to estimate local muscle oxygenation by measuring hemoglobin‐related variables and muscle oxygen saturation. This information helps understand how skeletal muscles respond to diverse types and intensities of resistance training, giving a clearer picture of metabolic activity during exercise. This scoping review aimed to examine the role of NIRS as a monitoring tool in resistance training and to synthesize existing evidence on its potential applications. A systematic scoping review was conducted across five specific electronic databases: PubMed, Scopus, Cochrane Library, SPORTDiscus, and Medline (EBSCO). A total of 1658 records were identified, with 78 studies included. Most focused on male participants and single‐joint exercises, especially knee extension. The vastus lateralis was most assessed, with isometric and isokinetic contractions predominating. Intensity was typically reported as %MVC or %1RM. About 42% used external interventions (e.g., supplementation, hypoxia, BFR). Continuous‐wave NIRS systems were most common, and methodologies varied widely across studies. NIRS in resistance training is emerging; it offers a noninvasive way to monitor internal load, but current evidence does not support its ability to predict hypertrophy or strength outcomes. Its practical relevance remains unclear and needs further investigation.

## 1 INTRODUCTION

Resistance training (RT) has been recognized for its numerous benefits and applications across different populations (Stojiljkovic et al., 2013). It promotes hypertrophy, enhances physical performance, and reduces the risk of injuries and diseases (Suchomel et al., 2016). In older adults, RT contributes to improved functional capacity and quality of life (Liu & Latham, 2009). Despite its widespread adoption, research continues to investigate its underlying mechanisms, individualized loading, and optimal training strategies. To advance understanding of RT and its physiological effects, researchers have developed various assessment tools. Accelerometers quantify barbell velocity during weightlifting exercises to evaluate power training (Sato et al., 2012). Additionally, physiological responses such as heart rate, lactate concentration, and oxygen saturation are measured to assess internal and metabolic load, adaptation, and fatigue during RT (Colosio et al., 2025; Grassi & Quaresima, 2016). Near‐infrared spectroscopy (NIRS) is a promising tool for assessing local muscle‐specific physiological responses during exercise. While primarily used to measure oxygenation and hemodynamic changes, its application in estimating internal load, defined as the physiological and psychological stress experienced by athletes during training or competition, remains under investigation. Additional studies are necessary to determine its validity and sensitivity for monitoring adaptation (Perrey et al., 2024).

NIRS is a noninvasive optical technique that measures local tissue oxygenation by emitting near‐infrared light, which penetrates several millimeters into biological tissue (Barstow, 2019). In skeletal muscle, the main light‐absorbing chromophores are hemoglobin (Hb) within the vasculature and myoglobin (Mb) within muscle cells, with a smaller contribution from mitochondrial cytochrome c oxidase (cyt ox) (Barstow, 2019). Because these chromophores change their absorption spectra depending on their oxygenation state, NIRS provides insight into the balance between oxygen delivery and consumption within the muscle (Drabkin, 1950; Ferrari et al., 2011; Hamaoka & McCully, 2019).

NIRS oximeters used to assess muscle oxygenation are classified into three main types: continuous wave (CW), time‐resolved (TRS or TD), and frequency domain (FD) (Ferrari et al., 2011). Among these, CW‐NIRS is the most widely used for its versatility and relatively low cost. However, its main limitation is that it only detects relative changes in chromophore concentrations, expressed in arbitrary units or percentage changes from a baseline, according to the modified Beer–Lambert law (Ferrari & Quaresima, 2012). CW systems typically provide two main output signals: a differential signal indicating tissue deoxygenation and a blood volume signal representing a total [Hb + Mb] (Ferrari et al., 2004). In contrast, FD and TD systems can measure absolute chromophore concentrations, such as oxygenated (O_2_Hb) and deoxygenated (HHb) hemoglobin (Ferrari & Quaresima, 2012).

A further source of confusion in the NIRS literature is the inconsistent terminology used to report tissue oxygenation outcomes. Terms such as muscle oxygen saturation (SmO_2_), tissue oxygen saturation (StO_2_), tissue saturation index (TSI), and tissue oxygenation index (TOI) are often used interchangeably across studies and devices, even though they are not always calculated or interpreted identically. These terms are frequently treated as synonyms, all calculated as a percentage reflecting the ratio of oxygenated hemoglobin to total hemoglobin (Barstow, 2019). However, the distinction between SmO_2_ and the broader term StO_2_ is often unclear and frequently conflated, in part because these terms do not explicitly reflect the layered, heterogeneous nature of the tissue beneath the probe (Feldmann et al., 2026). Two key factors differentiate SmO_2_ from StO_2_: the anatomical source of the optical signal, and whether the value represents a true oxygen saturation or a derived index (Feldmann et al., 2026). This distinction is largely conceptual rather than a fixed technical rule, since the labels used depend on the manufacturer and device rather than a standardized definition (Barstow, 2019; Feldmann et al., 2026). Consequently, values reported under different names may not be directly comparable across devices, and authors should specify the manufacturer‐defined outcome and its calculation method when reporting NIRS data. See Table 1.

**TABLE 1:** Mapping of common NIRS oxygenation terminology.

Term	Full name	Typical device type	Relationship to other terms
SmO_2_	Muscle oxygen saturation	CW (e.g., Moxy)	Intended as a muscle‐weighted estimate; often conflated with StO_2_ despite differing in anatomical specificity (Feldmann et al., 2026).
StO_2_	Tissue oxygen saturation	CW/FD	Broader, multi‐layer (skin/fat/muscle) signal; frequently used as a near‐synonym of SmO_2_ (Barstow, 2019; Feldmann et al., 2026).
TSI	Tissue saturation index	CW (e.g., Artinis)	Device‐specific naming convention, mathematically analogous to StO_2_ (Barstow, 2019).
TOI	Tissue oxygenation index	FD/CW	Used interchangeably with StO_2_/TSI in many studies; same general calculation principle (Barstow, 2019).

*Note*: To aid clarity, we adopt the term SmO_2_ (muscle oxygen saturation) throughout this review, regardless of the original terminology used in the included studies.

Despite its advantages, NIRS is influenced by several technical and biological factors. The signals can be affected by superficial tissues, muscle depth, and the presence of underlying bone (Hamaoka & McCully, 2019; Wassenaar & Van den Brand, 2005). In addition, muscle contractions alter tissue geometry and light scattering, increasing variability (Nourizadeh et al., 2025). These factors are important limitations that must be considered when interpreting NIRS data in research and applied contexts.

One of the most common applications of NIRS is to monitor muscle oxygenation during exercise. The advent of portable NIRS devices has increased accessibility, enabling researchers and practitioners to investigate physiological parameters that were previously difficult to measure (Perrey et al., 2024). During resistance training, NIRS allows real‐time monitoring of muscle oxygenation and hemodynamics, offering insights into the balance between oxygen delivery and utilization in active muscles. Studies have shown that muscle oxygen saturation (SmO_2_) can decrease substantially during working sets, from approximately 70% at rest to between 9% and 46% during maximal efforts (Miranda‐Fuentes et al., 2021). Furthermore, the test–retest reliability of NIRS during resistance exercises, such as the barbell squat, has been found to be acceptable, supporting its use in both applied and research settings (Corral‐Pérez et al., 2024).

This scoping review aims to provide a comprehensive overview of current applications of NIRS in assessing muscle oxygenation during resistance training. By synthesizing existing research, it seeks to clarify methodological approaches, highlight practical applications, and identify promising directions for future investigation.

## 2 METHODS

### 2.1 Protocol and registration

This scoping review protocol has been developed to systematically explore the use of NIRS to measure muscle oxygenation during RT. The review adheres to the PRISMA for Scoping Reviews (PRISMA‐ScR) guidelines to ensure methodological rigor and transparency (see Appendix S1). The protocol has been registered on the Open Science Framework OSF platform (DOI: 10.17605/OSF.IO/X342B), providing a public record of the planned methods and scope. This registration ensures the reproducibility of the review process and enables updates or amendments as the study progresses. All review stages, including the search strategy, screening, data extraction, and synthesis, are documented in accordance with the registered protocol.

### 2.2 Inclusion and exclusion criteria

This scoping review included full‐text, peer‐reviewed studies published in English that involved healthy, physically active adults aged 18–65 years, free from illness or injury. Eligible studies employed NIRS technology, whether Continuous Wave, Time Domain, or Frequency Domain, to measure changes in oxyhaemoglobin and deoxyhaemoglobin during resistance training. Studies were required to assess SmO_2_ using NIRS, either before and after an intervention or across sets.

All types of muscle contractions (concentric, eccentric, isometric, and isokinetic) were considered, provided the resistance training protocol required consistent effort across a full set of repetitions. Included protocols reflected traditional resistance training characteristics, encompassing both moderate‐ to high‐load training (≥60% 1RM) and low‐load resistance training (≤50% 1RM) performed to volitional failure or under blood flow restriction (BFR). The distinction between low‐load resistance training and endurance or aerobic protocols was based on the criterion of volitional muscular failure: protocols were included only when the level of effort and mechanical demand were sufficient to drive the muscle to failure within a discrete set, thereby inducing muscular fatigue consistent with resistance exercise rather than sustained aerobic work. This distinction is supported by recent evidence demonstrating that loads of 30%–90% 1RM performed to failure produce similar levels of local and whole‐body metabolic perturbation. In contrast, loads as low as 10% 1RM may not reach muscular failure and instead reflect an aerobic‐dominant effort (Colosio et al., 2026). Accordingly, protocols that did not target volitional failure, such as low‐intensity, prolonged, or submaximal continuous exercise primarily targeting aerobic or endurance adaptations were excluded, regardless of the absolute load used. Only studies conducted under normoxic oxygen levels were included. Studies involving hypoxia were considered only if hypoxia was part of a controlled experimental intervention.

### 2.3 Information sources and search

The literature search commenced on May 1, 2024, and continued until February 1, 2025. The study utilized various databases provided by the UQAM Library in the field of physical activity sciences, including PubMed, Scopus, SPORT Discus, Cochrane Library, Medline with full text (EBSCO), and ERIC (ProQuest), with the exclusion of ERIC due to its focus on education. Multiple keyword combinations were employed, and the selected combination adhered to the user guide on the Cochrane Library website: (Near‐infrared spectroscopy) OR (NIRS) OR (Oximetry) OR (Muscle oxygenation) OR (Tissue oxygenation) AND (Resistance training) OR (Resistance exercise) OR (Weight training) OR (Weight lifting) OR (Strength training) OR (Blood flow restriction) OR (BFR) OR (Ischemic preconditioning) OR (IPC). The results were screened to identify relevant studies by title, abstract, and finally by full text. Nonrelevant titles and abstracts were omitted.

### 2.4 Extracting data and selection criteria

Titles and abstracts of potentially relevant articles were screened by two reviewers MM and GA. Title duplicate publications were removed and articles that did not meet the inclusion criteria were excluded. Full texts were assessed for eligibility by authors and any articles that were ambiguous regarding inclusion criteria were excluded. Disagreements regarding inclusion of ambiguous articles were discussed with authors (MM and GA) and a consensus decision was taken with the help of a third reviewer. After the inclusion of an article in the review, relevant information from the article was systematically recorded in an Excel spreadsheet. The recorded information encompassed various attributes, such as the authors' names, publication date, participants' sex (Male and Female), anthropometric measurements, age, tools utilized, training modalities, specific exercises performed by participants, analyzed muscles, and the observed outcomes. When available, direct measures of muscle oxygenation, such as SmO_2_ (muscle oxygen saturation) and THb (total hemoglobin), were also documented. The recorded measures included preexercise values as well as those obtained after the completion of the last set, in cases where multiple sets were performed. Only studies that included at least one group performing resistance training under normoxic conditions without external interventions were considered and recorded in the spreadsheet.

## 3 RESULTS

### 3.1 Articles retained

**FIGURE 1:**
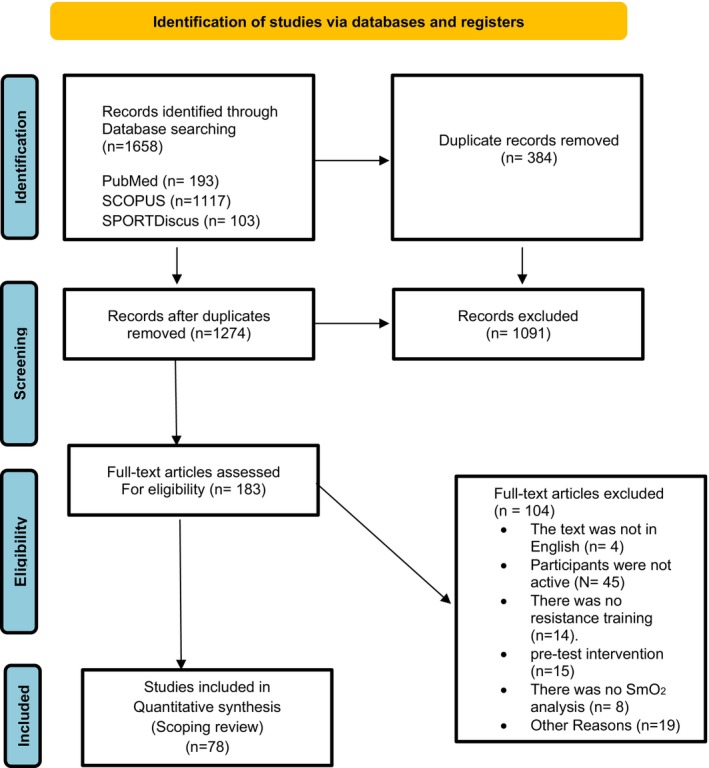
The PRISMA flow diagram of studies selection process.

A total of 1658 records were identified across four databases. After removing 384 duplicates, 1274 records were screened, and 1091 were excluded based on titles and abstracts. Full texts of 183 articles were assessed, with 105 excluded for reasons such as inactive participants, absence of RT, or missing SmO_2_ data. In total, 78 studies met the inclusion criteria and were included in the scoping review. The detailed screening process is illustrated in Figure 1.

### 3.2 Including studies and study characteristics

Among the 78 studies included in this review, participant characteristics and methodological approaches varied substantially. In terms of participant gender, 60 studies (77%) focused exclusively on males, 4 studies (5%) on females, and 14 studies (18%) included both sexes.

Exercise type varied across studies. Knee extension was the most frequently investigated exercise (40/78; 51%), followed by squats (8/78; 10%), bench press (5/78; 6%), and deadlift (2/78; 3%), reflecting the predominance of single‐joint lower‐limb protocols while still including complex, multi‐joint tasks.

Regarding NIRS measurement sites, the vastus lateralis (VL) was the most common muscle assessed (42/78; 54%), followed by the biceps brachii (10/78; 13%) and rectus femoris (9/78; 12%). These distributions indicate a research emphasis on major muscles of the upper and lower limbs relevant to resistance training tasks. With respect to muscle contraction type, isometric protocols were the most common (16/78; 21%), followed by isokinetic (14/78; 18%). Contraction intensity was typically reported as either a percentage of maximal voluntary contraction (26/78; 33%) or as a percentage of one‐repetition maximum (25/78; 32%).

### 3.3 External intervention

A total of 33 studies (42%) implemented an external intervention in addition to load manipulation. The most common interventions were supplementation and hypoxic conditions, each reported in 9 studies (12%). Blood flow restriction (BFR) and ischemic preconditioning (IPC) were examined in 6 studies each (8%), while lower body negative pressure (2/78; 3%) and cold‐water immersion (1/78; 1%) were less frequent.

Eighteen different NIRS devices were used across all studies. The continuous‐wave system dominated the field (71/78; 91%), with only a small number of studies using frequency‐domain (6/78; 8%) or time‐domain (1/78; 1%) devices. The main characteristics of the included studies are summarized in Table 2 below.

**TABLE 2:** Characteristics of the included studies.

Variable	Category	Number of studies (*n*)	% of Total (*n* = 78)
Participant Gender	Male only	60	77%
	Female only	4	5%
	Both sexes	14	18%
Exercise Type	Knee extension	40	51%
	Squat	8	10%
	Bench press	5	6%
	Deadlift	2	3%
NIRS Muscle Site	Vastus lateralis	42	54%
	Biceps brachii	10	13%
	Rectus femoris	9	12%
Contraction Type	Isometric	16	21%
	Isokinetic	14	18%
Force Measurement Method	%MVC	26	33%
	%1RM	25	32%
External Intervention	Supplementation	8	10%
	Hypoxia	10	13%
	BFR	9	12%
	IPC	6	8%
	Lower body negative pressure	2	3%
	Cold‐water immersion	1	1%
NIRS Technology	Continuous‐wave	71	91%
	Frequency‐domain	6	8%
	Time‐domain	1	1%

Among the NIRS devices, the Portamon device was the most frequently used, appearing in 23 studies. The NIRO device and its variants (200, 200×, 300, and 500) were the only other devices used in 10 or more studies, totaling 11 studies. Details of these devices are summarized in Table 3.

**TABLE 3:** NIRS Tools used in various studies presented in order of popularity.

NIRS tools	Types of waves	Numbers of studies	Native metric (units)
Portamon	Continuous wave	23	O_2_Hb, HHb, TSI (relative, arbitrary units/%)
NIRO (300;200;200×;500)	Continuous wave	11	O_2_Hb, HHb, TOI (relative, μM change/%)
Oxymon	Continuous wave	8	O_2_Hb, HHb, TSI (relative, arbitrary units/%)
Moxy	Continuous wave	6	SmO_2_, THb (relative, %)
Oxiplex TS	Frequency domain	5	O_2_Hb, HHb, StO_2_ (absolute, μM/%)
Inspectra	Continuous wave	4	StO_2_ (relative, %)
MicroRunman Nim Inc.	Continuous wave	3	O_2_Hb, HHb (relative, arbitrary units)
HB14 ASTEM	Continuous wave	3	O_2_Hb, HHb (relative, arbitrary units)
Nonin‐7600	Continuous wave	2	StO_2_ (relative, %)
ISS Imagent	Frequency domain	2	O_2_Hb, HHb, StO_2_ (absolute, μM/%)
Moor‐VMS‐NIRS: Moor instruments.	Continuous wave	2	O_2_Hb, HHb (relative, arbitrary units)
CW‐NIRS, NIMS philadelphia	Continuous wave	2	O_2_Hb, HHb (relative, arbitrary units)
Portalite	Continuous wave	2	O_2_Hb, HHb, TSI (relative, arbitrary units/%)
Prototype Ellerby	Continuous wave	1	O_2_Hb, HHb (relative, arbitrary units)
TRS‐20	Time domain	1	O_2_Hb, HHb, TOI (absolute, μM/%)
OM‐200	Continuous wave	1	O_2_Hb, HHb (relative, arbitrary units)
PIG‐200, OMRON	Continuous wave	1	O_2_Hb, HHb (relative, arbitrary units)
NIMo, nirox srl	Continuous wave	1	O_2_Hb, HHb (relative, arbitrary units)
Artinis not specified.	Continuous wave	1	O_2_Hb, HHb, TSI (relative, arbitrary units/%)

Table 4 summarizes the key characteristics of all 78 studies included in this review. It documents participant demographics, experimental methods (NIRS devices, muscles examined, exercise types), and measured outcomes. This consolidated format enables quick identification of research trends and methodological variations across studies, highlighting the heterogeneity in NIRS applications during RT research.

**TABLE 4:** Summary of participant characteristics and trial characteristics of included studies.

Authors	Participants	Exercise	Technology	Muscle(s)	NIRS objective	Methodology of training	Comments
Angleri et al. (2020)	12 M (Age: 23 ± 2; Height: 177 ± 0.3 cm; BM: 79 ± 5 kg)	Knee extension	Oxymon	VM	Observed if different training methods generate different responses at the NIRS data level.	TRAD: 3 sets of 10 reps at 75% 1RM; DS: 3 sets 75% 1RM to failure, followed by ~15 s rest and ~ 20% load drops, repeated twice within each set. PC: 3 sets of 10 reps at 75% 1‐RM, 8 reps at 80%, and 6 reps at 85%.	DS training produced lower levels of HbO_2_ and Hb Diff compared to TRAD and PC training, with no significant difference observed between TRAD and PC.
Ahmadi et al. (2008)	10 M (Age: 25 ± 4; Height:173 ± 7 cm; BM: 73 ± 9 kg)	Biodex elbow flexion	ISS oxyplex TS	Biceps	Eccentric exercise may decrease SmO_2_.	2 sets at 70 MVC (4 s of contraction, 12 of recovery)—eccentric contraction‐eccentric.	Maintaining a higher percentage of available HbO_2_ after muscle damage caused by exercise eccentric.
Akima and Ando (2017)	11 M (Age: 25 ± 7; Height: 174 ± 7 cm; BM: 71 ± 12 kg)	Isometric Knee extension	HB14 Astem	Quadriceps	Assess the SmO_2_ of the quadriceps muscles during muscle contraction.	50% MVC until failure.	Fatigue time was associated with VM and RF SmO_2_, but not VI or VL.
Alhammoud et al. (2018)	22 M (Age: 26 ± 4; Height:179 ± 4 cm; BM: 83 ± 6 kg)	Isokinetic knee extension	Portamon	VL	To assess the effects of hypoxia on the rate of development of the moment of strength.	35 max knee extensions@180°/s (90°–45°), 250 ms each, return@30°/s. Total: 61.25 s, arms crossed.	Hypoxia limits the rate of torque development and the rate of EMG rise; however, the change between maximal and minimal deoxyhemoglobin for each contraction was similar in hypoxia and normoxia, indicating that overall SmO_2_ was largely maintained despite hypoxic exposure.
Alvares et al. (2012)	13 M	Isokinetic concentric elbow extension	Microrunman Nim inc	Biceps	To assess the effects of L‐Arginine on biceps brachial strength and performance.	3 sets 10 reps MVC (60°s^−1^) 2 min rest.	Supplementation increases blood flow, but not SmO_2_ during recovery.
Alvares et al. (2020)	12 M	Isokinetic flexion and extension of the knee	Portamon	VL	To study the relationship between NIRS and the rate of contraction in RT.	2 methods: 1 set 6 slow reps (30°s^−1^); 1 set 6 quick reps (180°s^−1^).	NIRS‐derived total hemoglobin ([tHb]) reflects muscle blood flow during recovery from resistance exercise regardless of contraction velocity, indicating that [tHb] can be used to monitor perfusion responses at both slow‐ and fast‐velocity contractions.
Anders et al. (2021)	9 M (Age: 21 ± 2; Height: 181 ± 11 cm; BM:85 ± 6 kg)	Leg extension	Portamon	VL	Observed different NIRS data and different parameters.	50 reps 180°s^−1^ (Bilateral and unilateral)	NIRS revealed high intra‐ and interindividual variability in SmO_2_ during fatiguing leg extensions, with greater unilateral fatigue not explained by oxygenation differences.
Baláš et al. (2020)	34 (17 M.; Age: 27 ± 10) (17 F; Age: 26 ± 4)	Handgrip contractions	Oxymon	Deep flexors of the fingers	To assess whether different benefits emerge depending on the cold‐water immersion method and gender.	3×@80% MVC to fatigue, 20 min random recovery between.	Cold water immersion led to a lower SmO_2_ during contraction for both sexes.
Behrens et al. (2020)	16 M (Age: 26 ± 4; Height: 183 ± 6 cm; BM: 81 ± 8 kg)	Unilateral isometric knee extension	Moxy	VL	Assess whether the IPC improves performance.	20% MVT until fatigue	IPC has no effect on SmO_2_ during submaximal exercise.
Bhambhani et al. (2014)	11 (8 M,3 F) (Age: 34 ± 5)	Elbow flexion	Oxymon	Biceps	Observe the hemodynamic response of the muscle at different parameters.	2 min intermittent isometric contractions (12/min) at 20%, 40%, 60% MVC (90° elbow), 3 min rest between.	The decline in HbO2 suggests a greater need for SmO_2_ when the intensity increases.
Bloomer et al. (2010)	19 M (24 ± 4; Height 176 ± 5 cm; BM 80 ± 7 kg)	Bench press	Inspectra	Anterior Deltoid	Comparing glycine propionyl L‐carnitine to three other supplements and their effects on performance.	10 sets of 50% 1RM until failure. 2 min rest between.	GlycoCarn was the only supplement to show higher SmO_2_ at the start of exercise, indicating improved oxygen availability compared to other products. However, overall, none of the supplements enhanced blood flow or acute exercise performance.
Broxterman et al. (2015)	6 M (Age:25 ± 4; Height: 179 ± 4 cm; BM: 82 ± 10 kg)	Grip force	Oxyplex TS	Finger flexors	To investigate the relationship between central and peripheral fatigue and the curvature constant.	50% contraction intermittent (1.5 on, 1.5 off) 20 reps/min until failure.	BFR markedly increased both peripheral and central fatigue by restricting muscle oxygen availability and recovery. The findings suggest that reduced oxygen delivery under occlusion accelerates fatigue development and limits postexercise recovery.
Bunevicius et al. (2018)	8 M (Age: 22 ± 0.3)	Plantar flexion	Inspectra	Gastrocnemius Medial	To investigate the effects of occlusion on the late recovery period.	75% MVC 30 reps/min until failure.	Applying BFR late in the recovery period after high‐resistance exercise reduces SmO_2_ and impairs work capacity during subsequent high‐resistance exercise.
Cayot et al. (2016)	7 M (Age: 24 ± 1; Height: 181 ± 3 cm; BM: 81 ± 0.1 kg)	Isometric knee extension	Oxyplex TS	VL	Observe how the different occlusion levels affect SmO_2_.	1 set of 4 submaximal isometric knee extensions at 90° flexion was performed for each condition at 20%, 40%, 60%, and 80% MVC.	Applying blood flow restriction 5 min before exercise increased muscle deoxygenation (deoxy‐[Hb + Mb]) compared to immediate or no occlusion, indicating greater metabolic stress and reduced oxygen availability during low‐intensity exercise.
Cettolo et al. (2007)	10 M (Age: 28 ± 6; Height: 180 ± 10 cm; BM:77 ± 9 kg)	Leg press	Niro‐300	VL	Assess the SmO_2_ response according to the person's physical activity level.	5 × MVC(2–4 s) 2 min rest.	The VL muscle of people accustomed to RT may have a later activation of the oxidative metabolic system, or a greater amount of oxygen stored during a short and rapid maximum contraction isometric.
Davis et al. (2020)	11 (5 F; 6 M) (Age: 23 ± 1)	Squat	Moxy‐3	VL	Compare different energy demands between the front squat and the back squat.	2 exercises (front and back squat): 3 sets of 15 reps 70% 1RM 2‐3 min rest.	No difference in SmO_2_ when the two exercises are compared. However, SmO_2_ recovery is slower for the back squat.
Denis, Bringard, & Perrey (2011)	10 (9 M; 1 F) (Age:27 ± 3; Height:175 ± 7 cm; BM:66 ± 9 kg)	Knee extension	Niro‐300	VL	Assess whether eccentric training brings a greater decrease in SmO_2_ than concentric training.	Eccentric 60°s^−1^ concentric 60°s^−1^ until failure, “as hard as you can”.	The results suggest that a maximum repetition of an eccentric contraction causes greater extraction of O2 in the muscle, when compared to a concentric contraction.
Denis, Wilkinson, & De Vito (2011)	11 M (Age:25 ± 3; Height: 182 ± 6 cm; BM: 77 ± 6 kg)	Knee extension	Niro‐200	VL / RF	Investigated how angular velocity affects VL and RF SmO_2_.	Maximum contractions 10× 30°s^−1^ 60°s^−1^ 120°s^−1^ 240°s^−1^	The main results of this research are that the TOI of the VL and RF are significantly lower when contracted at 30° when compared to the other three methods.
de Oliveira et al. (2018)	12 M (Age:29 ± 9; Height: 137 ± 6 cm; BM: 81 ± 10 kg)	Isotonic grip strength	Portamon	Forearm flexor	To assess the effects of a beetroot supplement on muscle contraction, tHb and SmO_2_.	3× until fatigue 60 contractions/min (0.5 c; 0.5rest) followed by one‐minute rest.	Recovery of SmO_2_ in muscles was greater following beetrot gel supplementation.
De Ruiter et al. (2007)	15 M (Age:20–30; Height:183 ± 6 cm; BM:76 ± 6 kg)	Knee extension	Oxymon	VL/ RF VM	Investigated the knee‐extensor torque threshold for reoxygenation cessation, its relation to MTC, and differences among synergists.	The moment levels assessed were (randomized): 20%, 25%, 30%, 35%, 40%, and 45% MTC. 10 min rest between sets.	The reoxygenation of the superficial parts of the VL and VM muscles stops during a contraction of 25% MTC, however, for RF, it stops at 35% MTC.
De Ruiter et al. (2012)	17 M. (Age: 27 ± 7; Height: 183 ± 8 cm; BM:78 ± 9 kg)	Knee extension	Oxymon	VL/ RF /VM	To assess the moment of force threshold for peripheral fatigue during repeated isometric contraction.	5× 6 min from rhythmic contraction MVC (30%, 50%, 20%, 40%, 20%, 20%, 35%).	Observing EMG and muscle deoxygenation during submaximal contraction of knee extensors seems to be a feasible method for estimating the onset of peripheral fatigue for exercise related to aerobic capacity.
Downs et al. (2014)	13 (5 M 8 F) (Age:31 ± 12; Height: 169 + 12 cm; BM: 68 ± 12 kg)	Unilateral left leg press and heel raise exercise	Ellerby Prototype	VL	Assessed local vascular responses, (SmO_2_), and cardiovascular responses.	3× until voluntary fatigue 90 s of recoveries HL:80% 1RM LL: 20% 1RM LL‐BFRSBP: 20% 1RM/1.3× supine resting SBP.	LL‐BFR significantly reduces (SmO_2_) during exercise and impairs recovery postexercise, unlike LL and HL conditions, where SmO_2_ returns to baseline during rest
Formenti et al. (2018)	8 F (Age: 24 ± 4; Height: 164 ± 4 cm; BM: 63 ± 7 kg)	Knee extension	ISS imagent	VL	To assess the effects of speed during RT on SmO_2_.	3 visits of 3 series (2 s, 6 s, 10 s per contraction): contraction until fatigue, 3 min rest.	The slower speed of movement creates higher deoxygenation rates compared to normal speed movements.
Fryer, Stoner, Scarrott, et al. (2015)	29 M (9 intermediate) (Age: 29 ± 4; Height: 178 ± 9 cm; BM: 79 ± 1 kg), 10 Advanced (Age: 27 ± 5; Height: 179 ± 7 cm; BM: 71 ± 10 kg), 10 Elite (Age: 30 ± 9; Height: 175 ± 7 cm; BM: 69 ± 5 kg)	Grip strength	NONIN‐7600	FDP, FCR	To determine the hemodynamic kinetics of the forearm flexor muscles on climbers of three different levels.	40% MVC until voluntary fatigue.	Elite and advanced climbers have a greater capacity to deoxygenate the FDP and FCR muscles than intermediate climbers and nonclimbers during sustained contraction.
Fryer, Stoner, Dickson, et al. (2015)	44 M	Grip strength	NONIN‐7600	FDP, FCR	To assess the oxidative capacities of muscles for climbers of three different levels.	Up to voluntary fatigue: sustained and intermittent (10s on, 3 s off) 40% MVC.	Elite climbers have a faster recovery capacity to get to the recovery half zone than control and intermediate groups.
Ganesan et al. (2015)	6 M (Age: 25 ± 4; Height: 179 ± 4 cm; BM: 82 ± 10 kg)	Knee extension	TRS‐20 (TD)	VM	Use NIRS to measure the physiological responses of the VM during contraction with and without BFR.	3 sets until fatigue, 90 s recovery.	BFR during knee extensions increased muscle deoxygenation and reduced oxygen saturation, indicating higher metabolic stress and altered recovery kinetics.
Gepner et al. (2019)	10 M (Age: 21 ± 2; Height: 179 ± 4 cm; BM: 90 ± 11 kg)	Knee extension	Portamon	VL	Comparing SmO_2_ in young adults with middle‐aged during a protocol of isokinetic RT.	8 sets of 10 reps at 60°s^−1^, 3 min recovery.	Middle‐aged men showed lower SmO_2_ and higher deoxygenation than young adults during high‐volume resistance exercise, indicating age‐related reductions in local oxidative capacity.
Girard et al. (2019)	14 M (Age: 22 ± 1; Height: 179 ± 8 cm; BM: 72 ± 6 kg)	Barbell bicep curl, Lying triceps extension	Portamon Portalite	Biceps Brachii, Triceps Brachii	Quantifying the performance in a BFR procedure, systemic hypoxia, or both.	2 exercises (10 min between): 4 sets of 10 reps, 70% of 1RM, 90 s rest.	Markers of SmO_2_ and muscle activation were not affected by hypoxia, systemic or local, or a combination of both.
Gnimassou et al. (2018)	20 M (Age: 22 ± 0.4)	Knee extension	Portamon	VL	To determine the rates of protein synthesis with hypoxia or not.	8 sets of 8 reps at 80% 1RM, 2 min rest.	Acute hypoxia during resistance exercise did not increase short‐term muscle protein synthesis, and TSI decreased 10–15% during exercise with no difference between normoxia and hypoxia, indicating similar SmO_2_ responses.
Gomes et al. (2013)	31 (16 M and 15 F; Age: 28 ± 8; Height: 168 ± 6 cm; BM: 69 ± 11 kg)	Knee extension	Microrunman Nim Inc	VL	To determine whether hypoxia affects the SmO_2_ available during rest following a training.	Until muscle failure: Dynamic 50% or 75% 1RM (2 s concentric, 2 s eccentric), Static 100% 1RM.	The results demonstrate that short exposure to hypoxia is effective in reducing the oxygen available in the brain and muscles during resting conditions.
Gómez‐Carmona et al. (2020)	12 M (Age: 21 ± 1; Height: 181 ± 8 cm; BM: 77 ± 8 kg)	Squat	Moxy	VL	To assess the effects of strength training on SmO_2_ of the lower limbs.	Gradual increase over 6 sessions. Session 8 = 4 sets of 75% 1RM; 4 × 8 (10RM), 2 min recovery.	Higher effort levels and lower loads during resistance training lead to greater muscle deoxygenation, longer reoxygenation times, and increased fatigue, as measured by NIRS. SmO_2_ metrics effectively reflect skeletal muscle oxygenation and fatigue during exercise.
Goto et al. (2016)	16 M (Age: 21 ± 2; Height: 173 ± 5 cm; BM: 68 ± 9 kg)	Bench press (close grip)	HB14‐2 (ASTEM)	Triceps Brachii	Check the influence of different loads on muscle activity.	DS: series 1 (2% × 95% 1RM), series 2 (2% × 85% 1RM), series 3 (10% × 75% 1RM); Reverse DS: series 1 (3% × 55% 1RM), series 2 (3% × 65% 1RM), series 3 (10% × 75% 1RM).	DS causes greater SmO_2_ and intramuscular hypoxia than reverse drop‐set in people with RT experience.
Goto et al. (2019)	44 M (PRE (age: 21 ± 1; Height: 170 ± 3 cm; BM: 64 ± 5 kg), FRE (Age: 20 ± 0.9; Height: 169 ± 4 cm; BM: 63 ± 5 kg))	Elbow extension	HB14‐2 (ASTEM)	Triceps Brachii	To measure the short‐ and long‐term response of two training methods.	3 sets of 8 repetitions (8RM), 1 min rest.	Partial range of motion exercise produced greater decreases in oxygenated hemoglobin, indicating higher intramuscular hypoxia than full range of motion exercise. This greater hypoxic response was associated with enhanced muscle hypertrophy after 8 weeks of training.
Halley et al. (2018)	11 M (Age: 24 ± 3; Height: 176 ± 5 cm; BM: 77 ± 5 kg)	Knee extension	Portamon	Gastrocnemius	To measure the effects of IPC on neuromuscular fatigue.	Three 2‐min MVCs after IPC (3 × 5 min at 220 mmHg), SHAM (3 × 5 min at 20 mmHg), or control (30 min rest).	The IPC showed greater SmO_2_ in the contracting muscle, but without any evidence of improved performance (total work done).
Halley et al. (2019)	10 M (Age: 24 ± 3; BM: 80 ± 7 kg)	Leg extension	Moor VMS‐NIRS; Moor Instruments Ltd	RF	To determine whether IPC combined with dynamic exercise optimizes oxygen delivery to the muscle.	6 sets of 11 isokinetic knee extensions (120°/s ext., 300°/s flex.) over 70° ROM (starting at 90° flexion), at 40 reps/min. Each set lasted ~17 s with 20 s rest between.	IPC did not alter the SmO_2_ during the duration of the 3‐min exercise.
Hoffman et al. (2003)	11 M (Age: 20 ± 1; Height: 182 ± 7 cm; BM: 96 ± 14 kg)	Squat	cw‐nirs, NIMS, Philadelphia	VL	To examine the effects of two training protocols on SmO_2_ and anabolic hormonal response.	2 protocols (4 sets each): Protocol 1 = 15 reps at 60% 1RM, Protocol 2 = 4 reps at 90% 1RM.	The duration of the exercise has an influence on deoxygenation and the time of reoxygenation following an exercise.
Hoffman et al. (2007)	11 M (Age: 20 ± 1; Height: 182 ± 7 cm; BM: 96 ± 14 kg)	Squat	cw‐nirs, NIMS, Philadelphia	VL	To examine the effects of HL and LL RT on lipid peroxidation.	2 protocols: 5 sets of 15 reps at 60% 1RM, 5 sets of 4 reps at 90% 1RM.	The rate of reoxygenation following exercise is significantly correlated with the concentration of plasma malondialdehyde (MDA).
Jeffries et al. (2018)	20 M (Age: 26 ± 5; Height: 180 ± 6 cm; BM: 80 ± 12 kg)	Plantar Flexion	Portamon	Medial Gastrocnemius	Investigate IPC effects on oxidative capacity.	Submaximal isometric‐intermittent contraction of 40%–60% for 2 mins (2.5 s active/2.5 s rest).	Local adaptations potentiate oxidative functions and microvascular blood flow beyond 24–72 h of protection.
Nolan et al. (2020)	6 aerobic athletes (Age: 23 ± 1; BM: 77 ± 1 kg), 7 powerlifters (Age: 24 ± 1; BM: 80 ± 3 kg), 8 climbers (Age: 25 ± 2; BM: 74 ± 2 kg)	Grip force	Moxy	Superficial flexor of the fingers	Compare forearm flexors' isometric force.	3 protocols: (1) MVC to fatigue, (2) 40% MVC sustained for 3 min, (3) intermittent contraction for 3 min.	Climbers had greater resistance to contraction and lower O2 consumption during ischemic state in forearm flexors.
Kacin & Strazar (2011)	10 M (Age: 22 ± 0.6; Height:180 ± 2 cm; BM: 76 ± 3 kg)	Knee extension	Oxymon	VL	Understand whether doing Ischemic exercises with LL frequently improves SmO_2_.	4 sets of knee extensions to failure each session (4 times per week for 4 weeks) at 15% MVC, with one leg under ischemia and the other as control.	The NIRS results showed that the decrease in SmO_2_ during exercise was attenuated by 56% in the ischemic leg compared to 21% in the control leg, indicating enhanced muscle oxygen utilization and delivery following ischemic resistance training.
Keller & Kennedy (2021)	12 M (Age: 21 ± 3; Height: 184 ± 7 cm; BM: 85 ± 12 kg) 12 F (Age: 21 ± 0.9; Height:165 ± 3; BM: 71 ± 14 kg)	Force grip (biodex systems 3)	Portamon	Superficial flexors of the fingers	Examining the effects on SmO_2_.	25% MVC, until failure. (5% descent for 3 consecutive seconds).	Men desaturated faster than women and the observed difference in muscle mass, strength, and tissue are not the primary cause of the observed gender differences.
Keller et al. (2021)	15 M (Age: 21 ± 1; BM: 83 ± 10 kg) 15 F (Age:19 ± 0.8; BM: 64 ± 7 kg)	Knee extension	Portamon	VL	Examine the effects that sustained effort can have on SmO_2_ in men and women	MVC, until failure (5% descent for 3 consecutive seconds)	Females showed a slower decline in SmO_2_ than males, suggesting better oxygen maintenance during fatigue.
Kilgas et al. (2019)	10 M (Age: 27 ± 4; Height: 177 ± 0.1 cm; BM: 82 ± 14 kg).	Grip force	Oxymon	Forearm flexor	evaluated changes in blood flow and tissue perfusion before, during, and after exercise with BFR.	30 contractions, 30% MVC at 0%, 60%, 80%, 100%, and 120% of limb occlusion pressure.	The pressure of the occlusion turnstiles reduces the TOI and increases the concentration of HHb when it is at 60 and 80%.
Kojima et al. (2020)	25 M (Age: 24 ± 2; Height: 173 ± 5 cm; BM: 66 ± 7 kg).	Elbow flexion	Niro‐200	Biceps brachial (Long head)	To examine the acute effects of hyperoxia on dynamic muscular endurance and identify related individual factors.	30% 1RM/ Reps until failure.	Hyperoxia increased the number of repetitions and improved muscular endurance, accompanied by lower EMG amplitude and reduced muscle deoxygenation (higher Oxy‐Hb) during fatigue, indicating better muscle oxygen availability under hyperoxic conditions.
Kounoupis et al. (2022)	24 M (Age: 24 ± 4; Height:180 ± 6 cm; BM: 75 ± 8 kg).	Knee extension	Artinis (unspecified)	VL	Compare workload and vascular response.	Isometric 30% MVC for 2 min. Dynamic 30% MVC every 3 s for 2 min	Changes in O2Hb and HHb were larger for dynamic contraction, showing greater demand energy, when compared to isometric contraction.
Lin et al. (2014)	11 M (Age: 24. ± 3; Height:174 ± 7 cm; BM:64 ± 7 kg).	Knee extension	Imagent	VL	Compare muscle adaptations to SmO_2_ in sedentary and active people.	20% MVC/ 30s isometric	Trained people have a greater capacity to extract O2 than people who are not trained during muscle contraction.
Lockhart et al. (2020)	12 M (Age: 25 ± 5; Height:179 ± 6 cm; BM:81 ± 13 kg).	Knee extension	Portamon	VL	To determine whether the change in recovery time plays a role in physiological responses in hypoxia.	5 × 10 reps at 70% 1RM under 4 conditions: (1) hypoxia, 60 s rest, (2) normoxia, 60 s rest, (3) hypoxia, 180 s rest, (4) normoxia, 180 s rest.	Shortened interest rest during resistance exercise in hypoxia or normoxia increased muscle activation and perceived exertion, but did not alter vastus lateralis SmO_2_, heart rate, or quadriceps soreness.
Macleod et al. (2007)	11 M (Age: 22 ± 2; Height: 175 ± 6 cm. BM: 66 ± 6 kg).	Finger pressure	Niro‐500	Superficial flexors of the fingers	Determine several physiological responses through specific tasks to climbing.	Two isometric tests at 40% of MVC until volitional exhaustion: one with continuous contractions and one with intermittent contractions (10 s contraction, 3 s rest)	SmO_2_ during recovery is a good indicator of endurance performance
Marshall et al. (2020)	8 F (Age: 25 ± 5; Height: 168 ± 6 cm; BM: 71 ± 8 kg) 8 M (Age: 25 ± 6; Height: 179 ± 5 cm; BM: 86 ± 9 kg)	Knee extension	moorVMS‐NIRS; Moor	RF	Measure changes in fatigue and knee‐extensor torque	2 sets of 10 reps with maximum effort of 60°s^−1^	Similar reductions in muscle contractibility were observed for both sexes after training despite intersex differences in SmO_2_ during exercise isokinetic.
Martin et al. (2017)	15 M. (Age: 22 ± 2; Height: 179 ± 6 cm; BM: 84 ± 9 kg)	Knee extension	Portamon	VL	To examine whether a preworkout supplement enhances blood flow and SmO_2_ during resistance exercise at different loads.	4 sets until fatigue over 2 days, rhythmic 4.5 s per rep.1 day at 30% 1RM, 1 day 80% RM, 3 min recovery.	Lower minimum O_2_Hb at 80% load indicated greater muscle deoxygenation and metabolic stress, likely contributing to the enhanced hyperemic response with the preworkout supplement.
Guardado et al. (2020)	8 M 4 F (age: 27 ± 5; Height: 173 ± 10 cm; BM: 72 ± 13 kg)	Bench press	Moxy‐1	Pectorals	Compare perceptual, physiological, and technical responses including SmO_2_ between traditional and cluster resistance training protocols.	4 sets for 3 methods: (1) Training Traditional: 4 sets/6 repetitions 2 min rest. (2) Cluster 1: 4 sets of (3series of 2 reps separated by 15 s) 1 min 20 s of rest. (3) Cluster 2: 24 reps separated by 15 s between each	SmO_2_ remained similar across all protocols, indicating that cluster and traditional training produced comparable muscle oxygenation despite differences in fatigue and metabolic stress.
Matsuura et al. (2011)	11 M (Age: 29 ± 9; Height: 172 ± 4 cm; BM: 77 ± 6 Kg) 14 F (Age:27 ± 8; Height: 163 ± 4 cm; BM: 60 ± 8 kg)	Knee extension	Micro runman Nlm inc	VL	The examine the changes in cerebral and muscle blood volume and SmO_2_ during static and dynamic knee extensions to fatigue in men and women.	50%, 75%, or 100% 1RM to failure, followed by 4 min recovery. Dynamic trials (50%, 75%) involved reps from 90° to 180° at 2 s/phase, while static (100%) involved holding 180° until failure (≥10° drop).	Findings suggest that fatigue during resistance exercise (≤60 s) is primarily peripheral, driven by reduced blood volume and oxygen availability, regardless of contraction type, intensity, or gender.
Merrigan et al. (2020)	11 M (Age: 26 ± 1; Height:179 ± 9 cm; BM: 83 ± 9 kg)	Eccentric knee extension	Oxyplex TS	VL	To assess the effects of redistributing recovery time in shorter, but more frequent.	Traditional sets (TS): 4 × 10 with 95 s rest; Rest‐redistribution (RR): 20 × 2 with 15 s rest. Both protocols totaled 40 reps and 285 s of rest.	RR did not alter total muscle blood flow, but it achieved lower results in SmO_2_.
Mileva et al. (2006)	9 M (Age: 21 ± 3; Size:177 ± 7 cm, BM:70 ± 8 kg)	Knee extension	Niro‐500	VL	Assess whether the addition of vibration during knee extension influences performance.	4 sets of 8 reps (rhythmic 2.5 s per reps): 2% × 35% 1RM (with and without vibration) 2% × 35% 1RM (with and without vibration) 2% × 70% 1RM (with and without vibration).	The exercise of knee extension adds to a variable such as vibration stimulus causes residual changes in muscle activation and deoxygenation, resulting in improved mechanical performance.
Muñoz‐López et al. (2022)	30 M (Age: 21 ± 2; Height: 176 ± 7; BM: 74 ± 11 kg)	Squat	Niro‐200Nx	VL	Examine how different velocity loss (VL) thresholds affect muscle contractile properties and SmO_2_.	3 sets (60% 1RM) with 5‐min recovery: 20% VL vs. 40% VL.	Both velocity loss thresholds (VL20 and VL40) reduced SmO_2_ during the squat, but VL40 caused a slower deoxygenation rate in early sets and greater overall oxygen desaturation, indicating a longer period of reduced muscle oxygen supply compared to VL20.
Oliveira et al. (2017)	5 F (Age: 20–26)	Knee extension	Oxymon	RF	To assess the effects of Photo biomodulation on SmO_2_ and performance.	60 contractions concentric at 180°s^−1^	NIRS can be used as tools to measure the direct effects of photobiomodulation.
Oranchuk et al. (2020)	12 M (Age: 28 ± 8; Height: 176 ± 7 cm; BM: 75 ± 9 kg)	Knee extension	Moxy	VL	To compare SmO_2_ during voluntary and maximum muscle contraction with BFR or without.	60 s MVC with and without additional BFR	SmO_2_ during MVC was similar with or without additional BFR, indicating that sustained MVC alone induces near‐complete ischemia.
Paradis‐Deschênes et al. (2016)	10 M (Age: 25 ± 4; Height:177 ± 6 cm; BM: 85 ± 13 kg)	Knee extension	Portamon	VL	To examine the effects of IPC on hemodynamic response during maximal repetitions.	5 sets of 5 reps maximum at 20°s^−1^	3 cycles of IPC at 200 mm/Hg activate peripheral O2 consumption components and increase muscle perfusion at rest and recovery more than a placebo intervention (20 mm/Hg)
Paradis‐Deschênes et al. (2017)	9 M (Age: 25 ± 2; Height: 178 ± 2 cm; BM:86 ± 4 kg) 8 F (Age: 22 ± 1; Height: 166 ± 2 cm; BM: 60 ± 2 kg)	Knee extension	Portamon	VL	To determine the impact of IPC on muscle strength and hemodynamic response in men and women.	5 sets of 5 reps maximum at 20°s^−1^	Men with experience in RT may benefit more from the IPC than women during repeated and maximum repetitions.
Parganlija et al. (2019)	9 M (Age: 27 ± 5, Height: 181 ± 6 cm, BM: 80 ± 7 kg)	Leg press	Portamon	VL	Assess if SmO_2_ and performance are affected by simulated orthostasis.	2 methods (without pause; 4 s of pauses between each rep): 1 set of 15 reps at 60%1RM 4 s concentric—4 s eccentric	Negative lower body pressure superimposed on a slow concentric‐eccentric contraction can increase blood flow and SmO_2_ of the knee extensor muscles. This suggests an increase in muscle tissue perfusion.
Parganlija et al. (2020)	9 M (Age: 24 ± 4; Height: 178 ± 8 cm; BM: 74 ± 8 kg)	Leg press	Portamon	VL	Assess SmO_2_ and performance in simulated orthostasis.	1 set of 15 reps at 60% 1RM	Increased blood supply and oxygen utilization with negative pressure and slow contraction.
Penzer et al. (2016)	5 F (Age: 19–28; Height: 172 ± 8 cm; BM: 66 ± 9 kg)	Elbow flexion	Niro‐200	Brachial biceps	Compare effects of three training methods on SmO_2_.	3 methods (70% 1RM): (1) 3/7 (5 sets with repetitions increasing from 3 to 7 and short rest), (2) 4 sets of 6 reps and (3) 8 sets of 6 reps.	3/7 protocol showed greater SmO_2_ than others.
Piponnier et al. (2018)	24 M (Age: 21 ± 3; Height:179 ± 7 cm; BM: 72 ± 8 kg)	knee extension and plantar flexion	Portamon	VL and lateral gastrocnemius.	Comparing neuromuscular fatigue during MVIC between adults and children.	The intermittent voluntary fatigue protocol consists of 5 s of MVIC followed by 5 s of passive recovery until the moment of voluntary force reaches 60% of its initial value on three consecutive.	NIRS data showed that children experienced smaller decreases in SmO_2_ than men, indicating lower peripheral fatigue and reduced metabolic stress during repeated maximal efforts.
Piponnier et al. (2019)	22 M (Age: 21 ± 3; Height: 178 ± 7 cm; BM: 71 ± 8 kg)	Knee extension	Portamon	VL	Compare fatigue during MVIC in adult's vs. children.	Intermittent fatigue protocol: 5 s MVIC /5 s rest.	Children showed smaller decreases in SmO_2_ than men, indicating less peripheral fatigue.
Quaresima et al. (2001)	6 F (Age: 21)	Plantar flexion	OM‐200	Medial Gastrocnemius	Measure SmO_2_ during plantar flexion.	33% MVC for 1 min at 90–100° ankle angles, performed at 40, 60, and 80 cpm.	Medial gastrocnemius drives dynamic plantar flexion, with higher frequency/duration raising oxygen demand and femoral artery blood flow.
Reis et al. (2019)	13 M (Age: 23 ± 5; Height: 174 ± 4 cm; BM: 69 ± 7 kg)	Knee extension	NIMO, Nirox srl	VL	Measure physiological responses to different BFR intensities.	4 sets at 20% 1RM (30–15–15‐15 reps)	BFR hinders SmO_2_ during low‐load exercise.
Scott et al. (2017)	14 M (Age: 21–29; Height: 179 ± 5 cm; BM: 84 ± 11 kg)	Squat and deadlift	Portamon	VL	Assessing hypoxia effects on SmO_2_.	3 sets of 10 reps at 60% 1RM	SmO_2_ did not differ significantly in hypoxia vs. normoxia.
Scott et al. (2018)	12 M (Age: 25 ± 4; Height: 179 ± 4 cm; BM: 83 ± 9 kg)	Squat and deadlift	Portamon	VL	Assess physiological responses to hypoxia	5 sets of 5 reps at 80% 1RM under three conditions: normoxia, moderate hypoxia, and high hypoxia.	Hypoxia slightly affected SmO_2_, with significant decrease in SmO_2_ only during the deadlift, suggesting limited added benefit of hypoxia during high‐load resistance training.
Shadgan et al. (2009)	10 M (Age: 30 ± 6; Height: 177 ± 2 cm; BM: 79 ± 6 kg)	Force Grip	Portamon	Superficial flexors of the fingers	Evaluate NIRS tool for measuring chromophores	A series of 30 s sustained contractions at 10%, 30%, and 50% MVC, each followed by 3 min rest.	tHb and O_2_Hb significantly decreased during contraction, with greater reductions at higher contraction intensities.
Spiering et al. (2008)	9 M (Age: 25 ± 6; Height: 180 ± 6 cm; BM: 91 ± 10 kg)	Squat	Inspectra	Thigh muscle (unspecified)	Measure effects of L‐carnitine on SmO_2_.	4 sets of 15–20 reps (50% 1RM).	L‐carnitine supplementation reduced SmO_2_ during RT.
Stragier et al. (2019)	8 M and 8 F (Age: 24 ± 2; Height: 172 ± 9 cm; BM: 67 ± 13 kg), 7 M and 7 F (Age: 23 ± 2; Height: 172 ± 8; BM: 68 ± 11)	Elbow flexion	Niro‐200	Biceps brachii	Compare RT methods' physiological responses.	3/7 vs. 8 × 6 methods at 70% 1RM.	The 3/7 method induced greater fatigue and lower SmO_2_.
Thomas et al. (2020)	10 (5 M, 5 F) (Age: 19 ± 2; Height: 174 ± 5 cm; BM: 70 ± 6 kg)	Elbow flexion and knee extension	Niro‐200	Biceps brachii, RF	Characterize hemodynamic response in active/inactive limbs.	3 sets of 10 reps, 5 min rest.	SmO_2_ increase in active limbs during resistance exercise, while changes in inactive limbs were smaller and transient, highlighting localized oxygen delivery responses without evidence of vascular impairment.
Trepanowski et al. (2011)	13 M (Age: 23 ± 3; Height: 178 ± 8 cm; BM: 82 ± 12 kg)	Bench press	Inspectra	Anterior Deltoids	Examine the effects of 14 days of betaine supplementation on exercise performance and SmO_2_.	10 sets to fatigue (50% 1RM), 2 min rest.	Betaine supplementation led to greater decreases in SmO_2_ during bench press, suggesting increased muscle oxygen consumption.
Trexler et al. (2020)	27 M (Age: 22 ± 4; Height: 178 ± 6 cm; BM: 78 ± 12 kg)	Leg extension	Portalite	VL	Investigate the effects of citrulline malate (CitMal) and beetroot juice (BEET) on blood pressure, blood flow, and SmO_2_.	25% MVC for 3 min rhythmic contraction.	Neither CitMal nor BEET altered muscle oxygen consumption or muscle blood flow during submaximal resistance exercise.
Merrigan et al. (2020)	16 M (Age: 23 ± 2; Height: 181 ± 7 cm; BM: 81 ± 8 kg)	Knee extension	Oxiplex TS	VL	Assess effects of redistributing recovery time.	2 methods (60°s^−1^ and 360°s^−1^).	RR resulted in higher blood flow and SmO_2_ compared to traditional method.
Walden et al. (2020)	20 M (Age:23 ± 1; Height:178 ± 7 cm; BM:77 ± 11 kg)	Bench press and seated shoulder press	Portamon	Lateral head of the triceps of the nondominant arm	To assess whether hypoxia exchanges physiological responses and whether it accentuates changes when a series is performed at most against sub maximum.	2 Methods: DS involved a 10RM set followed by drops to 80% and 60%; DS‐between sets used two 10RM sets, each followed by the same 100%–80%–60% drop sequence.	The oxygen saturation index indicates that SmO_2_ was lower in hypoxia than in normoxia, for both methods.
Yamada et al. (2003)	9 M (Age: 19–21; Height: 172 ± 4 cm; BM: 64 ± 5 kg)	Knee extension	HEO‐200, OMRON	VM	Observing the relationship between SmO_2_ and contraction.	Isometric: 10 s at 30% MVC and 10 s at 50% MVC.	Muscle strength and muscle thickness are negatively correlated with SmO_2_.
Yokoi et al. (2014)	11 M (Age: 18–21; Height: 170 ± 5 cm; BM: 63 ± 12 kg)	Knee extension	Niro‐200	RF	Aimed to assess the effects of normobaric hypoxia on the recovery of fatigued muscle.	70% MVIC until fatigue.	Results suggest that 30% normobaric hyperoxia enhances recovery from acute muscle fatigue more effectively than normoxia.

Abbreviations: BM, Body Mass; CPM, Contractions Per Minute; CSA, Cross‐Sectional Area; DS, Drop Set; F, Female; FCR, Flexor carpi radialis; FDP, Flexor digitorum profundus; FRE, Full Range of Motion Exercise; HL, High Load; IPC, Ischemic Preconditioning; LL, Low Load; M, Male; MTC, Maximal Torque Capacity; MVC, Maximal Voluntary Contraction; MVIC, Maximal Voluntary Isometric Contraction; MVT, Maximal Voluntary Torque; PC, Crescent Pyramid; PRE, Partial Range of Motion Exercise; RF, Rectus Femoris; SmO_2_, Muscle oxygen saturation; TOI, Tissue Oxygenation Index; TRAD, Traditional Set; VI, Vastus Intermedius; VL, Vastus Lateralis; VM, Vastus Medialis.

### 3.4 Summary of main objectives of the studies

Table 5 presents the primary research objectives and categories identified in the reviewed NIRS studies. These studies are organized by experimental focus and comparative design, illustrating the application of NIRS in assessing muscle oxygenation and hemodynamic responses across various conditions.

**TABLE 5:** Summary of study objectives identified in the review.

Objectives of the study	Number of studies
Hypoxia	10
BFR	9
Supplements	8
IPC	6
Effect of fitness level	6
Contraction speed comparison	4
Age comparison	3
Sex comparison	3
Typical contraction comparison	3
Orthostasis	2
Vibration	1
Range of motion	1
Comparison between two movements	1
Comparison of unit versus bilateral contraction	1
Maximum force moment comparison	1
Ice water immersion	1
Comparison of responses of several muscles	1

Twenty‐five studies investigated external modulation factors, including BFR, supplementations (e.g., betaine, L‐carnitine), and hypoxia. These studies compared neutral controls with altered physiological states to assess changes in oxygen delivery and extraction. Seven descriptive studies characterized SmO_2_ responses during specific exercise tasks using a single intervention without a comparative condition. Ten studies compared training methods, such as traditional resistance, interval‐based, and BFR training protocols, to evaluate the effects of exercise volume, intensity, and rest intervals on deoxygenation and reoxygenation. Eleven studies examined participant characteristics, analyzing differences in NIRS responses by sex, age, training status (trained vs. untrained), and activity level (active vs. inactive), and athletic level to elucidate intrinsic variations in oxygen utilization. Five studies assessed the impact of contraction speeds and load intensities, demonstrating that faster or heavier efforts result in greater transient deoxygenation and highlighting NIRS sensitivity to workload stress. Additionally, several studies investigated reoxygenation and deoxygenation dynamics, employing SmO_2_ kinetics as indicators of oxidative capacity and microvascular function. Collectively, these findings reinforce NIRS as a robust, noninvasive method for monitoring muscle metabolic efficiency across a range of exercise conditions.

## 4 DISCUSSION

This scoping review systematically mapped the existing literature on the use of NIRS to monitor muscle oxygenation during RT. The findings indicate that NIRS technology shows strong potential as a noninvasive method for assessing local physiological responses to resistance exercise (Bhambhani et al., 2014). While current evidence mainly supports its use in monitoring acute muscle oxygenation changes, further research is required to confirm its effectiveness in tracking internal load, metabolic stress, and long‐term adaptations. Real‐time monitoring of muscle SmO_2_ and total hemoglobin (THb) enables detailed analysis of the relationship between oxygen delivery and utilization in active muscles. This approach may contribute to a better understanding of the mechanisms underlying fatigue development and could support future efforts to optimize training protocols aimed at improving hypertrophy and strength outcomes (Angleri et al., 2020).

A central finding of this review, which aligns with conclusions from individual studies and previous narrative reviews (Miranda‐Fuentes et al., 2021), is the substantial heterogeneity observed across the 78 included studies. Although the research objectives are broad and varied (see Table 4), the studies can be classified into four main categories. These categories clarify the contexts in which the use of NIRS is most relevant within resistance training research.

### 4.1 Observation of physiological responses during muscle contraction

The first category includes studies that used NIRS to assess physiological responses during muscle contraction, with an emphasis on identifying thresholds or muscle‐specific patterns. The earliest study reviewed (Quaresima et al., 2001) reported rapid desaturation of the medial gastrocnemius at approximately 33% of MVC during plantar flexion. Similarly, (Akima & Ando, 2017) compared deoxygenation patterns among quadriceps muscles during knee extension and found that fatigue‐related declines in SmO_2_ were most pronounced in the vastus medialis and rectus femoris. The authors noted that the vastus lateralis and vastus intermedius together comprise about 60%–65% of total quadriceps volume, which may explain observed intermuscle differences (Sattler et al., 2014). The vastus lateralis (VL) is often chosen as the primary site for NIRS‐based muscle oxygenation assessment due to its anatomical suitability for high‐quality signal acquisition. Compared to other muscles, the VL typically produces lower signal noise and more consistent measurements because of its uniform thickness, superficial location, and minimal interference from subcutaneous fat or nearby bone. As a result, research consistently identifies the VL as a reliable site for sensor placement in both static and dynamic exercise protocols (Koga et al., 2017; Miyamoto et al., 2020).

Several studies have also examined multi‐joint movements. One study found no significant differences in NIRS‐derived variables between back and front squats, indicating that front squats may create similar physiological demands even with lighter external loads. However, recovery reoxygenation was slower after back squats (Davis et al., 2020). Similarly, another study reported that effort level, defined as the proportion of completed repetitions relative to the maximum possible, was a stronger determinant of muscle desaturation than absolute load (Gómez‐Carmona et al., 2020). Other investigations have explored torque‐related oxygenation thresholds, finding that desaturation began at higher torque levels in the rectus femoris (35% MTC) than in the vastus medialis or vastus lateralis (25% MTC) (De Ruiter et al., 2007). In a subsequent study, performing submaximal knee extensor contractions while simultaneously monitoring EMG and muscle deoxygenation provides a practical approach for estimating an aerobic capacity‐related exercise intensity at which peripheral fatigue begins to develop (De Ruiter et al., 2012).

These results suggest that NIRS reliably measures physiological responses, oxygenation thresholds, and muscle‐specific behaviors during resistance exercise, though the underlying mechanisms require further study.

### 4.2 Comparison between different types of individuals

The second group of studies compared muscle oxygenation responses across different populations. Research on sex‐based differences consistently found faster muscle desaturation in men than in women (Keller et al., 2021; Keller & Kennedy, 2021; Marshall et al., 2020). These differences are attributed to women's higher capillary density (Roepstorff et al., 2006), greater tolerance to high‐intensity exercise, and enhanced vasodilatory responses during knee extension (Parker et al., 2007). Similar results were seen in Ischemic Preconditioning (IPC) studies, where women showed a reduced IPC response (Paradis‐Deschênes et al., 2017). Notably, research reported that although women had smaller reductions in muscle oxygenation, performance outcomes did not differ between sexes. Overall, current evidence, though limited, suggests that NIRS can detect sex‐related physiological differences during resistance exercise, but the mechanisms behind these differences remain unclear (Keller et al., 2021).

It is critical to note, however, that a major confounding factor in sex‐based and fitness‐status comparisons is the inconsistent reporting or control of Adipose Tissue Thickness (ATT), which acts as a primary source of systematic error across the literature. From a methodological standpoint, if the ATT at the measurement site is greater than half the distance between the NIRS light source and the detector, little or no signal from the underlying skeletal muscle will be detected (Stuer et al., 2025). Because near‐infrared light follows a curved migratory path through tissue, an uncorrected thick subcutaneous fat layer absorbs and scatters photons before they can interact with the active muscle bed, potentially fabricating false metabolic differences between sexes and populations.

Age‐related comparisons reveal distinct oxygenation patterns. Evidence shows that older adults (47 years) experience greater decreases in SmO_2_ than younger adults (21 years), with disparities increasing across sets. While deep desaturation in athletes reflects superior mitochondrial extraction capacity, in older adults it instead reflects impaired oxygen delivery, driven by age‐related vascular stiffness and reduced capillarization, which restricts blood flow to the muscle rather than enhanced extraction (Gepner et al., 2019). Additional research indicates that children display smaller desaturation responses than young adults, even when performing a similar number of repetitions (Piponnier et al., 2018, 2019). These studies suggest that fatigue in children may be more centrally mediated, reflecting lower muscular maturation, such as reduced capillarization and intracellular metabolic processes.

Sex‐related differences in SmO_2_ responses remain inconsistent in the literature. Although females generally exhibit greater capillary density and mitochondrial oxidative function than males, which would theoretically support greater oxygen delivery and extraction capacity (Bailleul et al., 2025), NIRS studies during fatiguing tasks have reported slower rates of desaturation in females compared to males (Keller & Kennedy, 2021), while resistance‐training‐specific studies have found no significant sex differences in SmO_2_ dynamics across sets (Claude [Internet], 2026). These conflicting findings suggest that sex‐related physiological advantages in oxidative capacity may not consistently translate into measurable differences in NIRS‐derived oxygenation during resistance exercise, warranting further investigation.

Training status and sport‐specific specialization were also shown to influence muscle oxygenation. Evidence suggests that trained individuals exhibit a delayed onset of desaturation during maximal isometric contractions compared to untrained participants (Cettolo et al., 2007). In climbing athletes, studies have demonstrated greater oxygen extraction in elite performers (Fryer, Stoner, Dickson, et al., 2015), and faster reoxygenation kinetics during recovery (Fryer, Stoner, Scarrott, et al., 2015). Additional research further indicates that climbers exhibit superior forearm oxygenation patterns relative to powerlifters and endurance athletes, highlighting sport‐specific adaptations in intracellular oxygen utilization (Nolan et al., 2020). Conversely, sedentary individuals display reduced O_2_ extraction capacity (Lin et al., 2014).

Although available studies are limited, current evidence indicates that NIRS is a reliable tool for identifying differences in muscle oxygenation during resistance exercise. Findings show that higher‐performing or more highly trained individuals generally demonstrate greater oxygen extraction and faster reoxygenation, especially during longer or repeated exertion.

### 4.3 Comparison between training methods

The third group of studies compared physiological responses across resistance training methods. Studies that manipulated training volume distribution found greater desaturation during drop sets than with traditional or reverse drop‐set protocols and during the 3/7 method compared to traditional loading (Angleri et al., 2020; Goto et al., 2016). Redistributed rest training resulted in greater desaturation compared to conventional sets (Merrigan et al., 2020). However, other studies reported no significant differences between these methods (Guardado et al., 2020; Matsuura et al., 2011). Collectively, these findings indicate that NIRS can differentiate hemodynamic demands among various training strategies. Comparative research on training loads demonstrates that higher loads increase oxygen demand and cause greater reductions in muscle oxygenation (Bhambhani et al., 2014; Shadgan et al., 2009; Yamada et al., 2003), likely as a result of elevated intramuscular pressure. Hoffman et al. also reported that lower relative intensities (60% 1RM) are associated with longer reoxygenation delays compared to higher intensities (90% 1RM), a phenomenon potentially attributable to the Bohr effect (Hoffman et al., 2003). Additionally, oxidative stress, as indicated by malondialdehyde (MDA) levels, may further impair reoxygenation (Hoffman et al., 2007). These findings collectively suggest that training load significantly affects muscular oxygen extraction.

Contraction of velocity influences oxygenation, as slower contractions consistently result in greater desaturation (Alvares et al., 2020; Formenti et al., 2018). Similarly, velocity‐loss‐based training demonstrates that larger velocity losses are associated with greater desaturation (Muñoz‐López et al., 2022), which aligns with the established finding that slower velocities necessitate increased motor‐unit recruitment (Barnes, 1980).

Studies comparing contraction type showed higher HbO_2_ following eccentric contractions (Ahmadi et al., 2008) and greater desaturation during eccentric versus concentric actions during maximal efforts (Denis, Bringard, & Perrey, 2011). Dynamic contractions also imposed greater energetic demand than isometric contractions at matched intensity and volume (Kounoupis et al., 2022), consistent with earlier evidence of higher activation costs (Chasiotis et al., 1987). Overall, these studies highlight metabolic stress, intramuscular pressure, and contraction velocity as key determinants of muscle desaturation during resistance training, reinforcing their potential relevance for hypertrophic adaptations.

Despite these findings, direct comparison across studies remains challenging due to two underreported issues. First, the reproducibility of NIRS during resistance training is rarely quantified; a few studies report trial‐to‐trial or day‐to‐day reliability metrics, which limit confidence in the consistency of the reported responses (Hamaoka & McCully, 2019). Second, spatial heterogeneity within large muscles means that a single probe placed over the vastus lateralis or rectus femoris may not represent whole‐muscle oxygenation, as oxygenation can differ substantially between measurement sites on the same muscle (Hamaoka & McCully, 2019; Jones et al., 2016). Multichannel NIRS approaches and standardized probe placement protocols are needed to reduce this variability in future research.

Quantified reproducibility data remain scarce in the resistance training NIRS literature, but device‐specific evidence from exercise studies provides useful benchmarks. For the Portamon (CW), within‐session reliability for SmO_2_ maximum and minimum has been reported as good to excellent, whereas between‐session reliability is substantially lower, with ICC values as low as 0.53 for some parameters (Desanlis et al., 2022). Interday test–retest reliability for resting TSI in the Portamon showed a CV of 1.8%–2.5%, compared to 5.7%–6.2% for the Moxy (McManus et al., 2018), indicating that the Portamon is more stable across sessions at rest. During exercise, SmO_2_ ICC for the Portamon ranged from 0.81 to 0.90 across exercise intensities, with SEM values of 5%–7% and a minimal detectable change of 12%–18% (Yogev et al., 2023). For the NIRO‐200Nx (CW), reliability during squat exercise showed good relative reliability, with ICC values of 0.851–0.899 for O_2_Hb and HHb, and CV for TOI ranging from 2.7% to 10.2% (Corral‐Pérez et al., 2024). Regarding device comparisons, the Moxy and Portamon report similar TSI values only at low adipose tissue thickness (ATT <7 mm), with differences increasing to −14.7% ± 2.8% at ATT >10 mm, largely due to the smaller source–detector separation in the Moxy (McManus et al., 2018). When comparing CW and FD technologies, a wearable CW device showed strong correlation with an FD reference system (*r* = 0.792), with 95% of measurements falling within ±8% SmO_2_ (Peikon et al., 2025), suggesting acceptable agreement for field use, though absolute values should not be used interchangeably across technology types.

### 4.4 External interventions

The primary objective of this group of studies was to determine whether NIRS‐derived variables are influenced by external interventions, rather than solely observing oxygenation responses during exercise.

Hypoxia is broadly defined as a reduction in tissue oxygen availability, whether defined as decreased tissue oxygen (Gnimassou et al., 2018) or reduced inspired oxygen fraction (Scott et al., 2018) influence local muscle oxygenation. Previous research has examined this issue under various conditions, including altered oxygen availability (Alhammoud et al., 2018; Gomes et al., 2013; Walden et al., 2020), consistently showing that systemic hypoxia is detectable at the local muscle level via NIRS.

BFR and IPC utilize controlled occlusion to induce acute physiological adaptations. Since NIRS provides an indirect estimate of blood flow (Neary, 2004) and is particularly sensitive to interventions that alter local perfusion. Studies employing NIRS during BFR (Broxterman et al., 2015; Bunevicius et al., 2018; Cayot et al., 2016) consistently identify increased muscle deoxygenation as a central acute response, even when performance outcomes remain unchanged. For example, Oranchuk et al. (2020) observed no performance difference between BFR and maximal voluntary isometric contraction, highlighting the role of intramuscular pressure in regulating blood flow during occlusion‐based interventions.

Nutritional supplementation studies have explored whether it influences performance by modifying muscle oxygenation. L‐arginine increased local blood flow, yet SmO_2_ and strength performance remained unchanged, suggesting no meaningful alteration in muscle oxygen extraction (Alvares et al., 2020). Beetroot juice and caffeine supplementation were associated with higher SmO_2_ values during early exercise, indicating a potential effect on oxygen delivery in the initial phases of effort (Bloomer et al., 2010; Trepanowski et al., 2011). Other supplements, including creatine and beta‐alanine, produced no detectable changes in NIRS‐derived variables (Martin et al., 2017), while certain supplements appeared to influence reoxygenation kinetics during recovery rather than during the exercise bout itself () also reported NIRS‐detectable changes in muscle oxygenation alongside alterations in performance.

Overall, this body of research demonstrates that muscle oxygenation during resistance exercise is sensitive not only to contraction characteristics and exercise intensity but also to a wide range of external factors. NIRS appears to be a valuable tool for detecting and quantifying the acute physiological effects produced by these interventions.

Future research should explore whether it is possible to establish athletic profiles or predict performance based on the degree of muscle desaturation and the rate of reoxygenation. Hypertrophy is a recurring and promising theme in the current literature, and it would be valuable to determine whether tailoring training methods according to NIRS‐derived parameters could enhance hypertrophic outcomes. Faster reoxygenation appears to reflect an individual's endurance capacity, and findings indicate slower recovery after front squats compared with back squats (Davis et al., 2020) raise the possibility that reoxygenation speed may also serve as an indicator of relative exercise intensity within the same individual.

More broadly, future studies would benefit from standardizing key methodological elements to improve comparability across research. The physiological calibration framework proposed by Jones et al. (2016), along with the checklist developed by Perrey and Ferrari, may help reduce discrepancies between NIRS devices and enhance the reliability of measurements (Perrey & Ferrari, 2018).

### 4.5 Limitations and strengths

One limitation of this review is the diversity among the studies that have evaluated RT and NIRS tools. This is evident in the wide variation in objectives, muscles assessed, NIRS tools utilized, types of athletes, coaching methods, and more. As a result, direct comparisons of the results become challenging. Another limitation is that a scoping review, being a summary of studies, does not incorporate an assessment of the risk of bias in the included studies. Nonetheless, the substantial number of articles, recent publication dates of the selected studies, and their summaries offer an overview of the current state of research in this field.

Several strengths characterize this review. The search strategy employed was both rigorous and comprehensive. Additionally, the structured organization of internal and external training variables provides a clearer framework for interpreting diverse findings. Combined with the large number and recent publication dates of the included studies, these strengths contribute to an up‐to‐date and well‐organized overview of the current research landscape.

## 5 CONCLUSIONS

Research employing NIRS in resistance training remains in an early phase, and this review highlights both its potential applications and the current limitations in understanding. While NIRS provides a noninvasive means of quantifying internal load, its relevance for predicting hypertrophy or strength outcomes is not yet supported by physiological evidence. Currently, it is unclear which NIRS‐derived variables beyond their descriptive value may meaningfully inform training prescriptions or performance assessments in RT. These uncertainties underscore the need for rigorous future investigations to determine the specific context in which NIRS can contribute to evidence‐based practice.

## AUTHOR CONTRIBUTIONS

**Moath Mohammad:** Conceptualization; data curation; methodology; resources. **Ghydaa Alshanty:** Conceptualization; data curation. **Juliette Langelier:** Conceptualization; data curation. **Morgane Mary‐Pouliot:** Conceptualization; data curation. **Michael Stolberg:** Conceptualization; data curation. **Rami Hammad:** Conceptualization; data curation. **Alain Steve Comtois:** Conceptualization; methodology; supervision.

## FUNDING INFORMATION

This work was supported by the Canadian Space Agency [grant number: 21FAQAMA07].

## CONFLICT OF INTEREST STATEMENT

The authors declare they have no conflicts of interest.

## ETHICS STATEMENT

Not applicable. This study is a scoping review and does not involve any direct interventions on human participants or animals. All data were obtained from previously published literature.

## Supporting information


**Appendix S1:** PRISMA‐ScR Checklist.

## Data Availability

All data underlying the findings of this systematic mapping and scoping review are contained fully within the manuscript and its Supporting Information (including the attached PRISMA‐ScR checklist and data tables).
