# Supplementary material for: Muscle oxygenation measured by near‐infrared spectroscopy during resistance training: A scoping review
Source: Physiol Rep. 2026 Jul 14;14(14):e71014. doi: 10.14814/phy2.71014 (PMC13369300; doi:10.14814/phy2.71014)
Supplement: Supplementary file 1 — Appendix S1: PRISMA‐ScR Checklist. [file PHY2-14-e71014-s001.docx]

**Supplementary files**

| **SECTION** | **ITEM** | **PRISMA-ScR CHECKLIST ITEM** | **REPORTED ON PAGE #** |
| --- | --- | --- | --- |
| **TITLE** | | | |
| Title | 1 | MUSCLE OXYGENATION MEASURED BY NEAR-INFRARED SPECTROSCOPY DURING RESISTANCE TRAINING: A SCOPING REVIEW. | Title |
| **ABSTRACT** | | | |
| Structured summary | 2 | This scoping review examined the use of near-infrared spectroscopy (NIRS) to monitor muscle oxygenation during resistance training. Seventy-eight studies were included, primarily involving male participants and single-joint lower-limb exercises, with the vastus lateralis most frequently assessed. Training intensity varied, and 42% of studies incorporated external interventions such as BFR, supplementation, or hypoxia. Continuous-wave NIRS devices were most used, though study methodologies were highly heterogeneous. While NIRS shows promise for monitoring internal load, current evidence does not support its use for predicting hypertrophy or strength, emphasizing the need for further research to clarify its practical applications. | Abstract |
| **INTRODUCTION** | | | |
| Rationale | 3 | Near-infrared spectroscopy (NIRS) is a non-invasive tool for monitoring local muscle oxygenation and hemodynamics, providing insight into internal load—the physiological and psychological stress during training. It measures changes in hemoglobin, myoglobin, and cytochrome c oxidase, with devices classified as continuous-wave (CW), time-resolved (TRS/TD), or frequency-domain (FD); CW systems are most common but measure only relative changes. NIRS signals are influenced by tissue thickness, muscle depth, bone, and contraction-induced geometry changes. Portable devices allow real-time monitoring during resistance training, with SmO₂ decreasing substantially during sets, and reliability is acceptable for research and applied use. | Introduction (Background) |
| Objectives | 4 | This scoping review aims to provide a comprehensive overview of current applications of NIRS in assessing muscle oxygenation during resistance training. By synthesizing existing research, it seeks to clarify methodological approaches, highlight practical applications, and identify promising directions for future investigation. | Introduction (Objectives) |
| **METHODS** | | | |
| Protocol and registration | 5 | This scoping review follows PRISMA-ScR guidelines to systematically examine NIRS use in monitoring muscle oxygenation during resistance training. The protocol is registered on OSF (DOI: 10.17605/OSF.IO/X342B), ensuring transparency, reproducibility, and documentation of all review stages, including search, screening, data extraction, and synthesis. | Methods (search strategy) |
| Eligibility criteria | 6 | This review included full-text, peer-reviewed studies in English with healthy adults (18–65 years). Eligible studies used NIRS (Continuous Wave, Time Domain, or Frequency Domain) to measure oxy- and deoxyhemoglobin changes and SmO₂ during resistance training. All contraction types were included, with protocols reflecting moderate- to high-load (≥60% 1RM) or low-load (≤50% 1RM) training to volitional failure or under BFR. Studies on aerobic/endurance exercise were excluded. Only normoxic conditions were included, with hypoxia considered solely in controlled interventions. | Methods (Selection of Study) |
| Information sources and Search | 7 | The literature search was conducted from May 1, 2024, to February 1, 2025, using databases in physical activity sciences, including PubMed, SCOPUS, SPORT Discus, Cochrane Library, and Medline (EBSCO). ERIC was excluded due to its educational focus. Multiple keyword combinations related to NIRS, muscle oxygenation, resistance training, BFR, and IPC were used. Results were screened by title, abstract, and full text, with non-relevant studies excluded. | Methods (Information sources and Search) |
| Extracting data and Selection Criteria | 8 | Titles and abstracts were independently screened by two reviewers (MM and GA), with duplicates and studies not meeting inclusion criteria excluded. Full texts were assessed for eligibility, and disagreements were resolved by consensus with a third reviewer. Data from included studies were systematically extracted into an Excel spreadsheet, recording details such as authors, publication year, participant characteristics (sex, age, anthropometrics), NIRS tools, training modalities, exercises, analyzed muscles, and outcomes. Muscle oxygenation measures (SmO₂ and THb) were documented, including pre- and post-exercise values. Only studies with at least one group performing resistance training under normoxic conditions without external interventions were included. | Methods (Data extraction and analysis) |
| **RESULTS** | | | |
| Selection of sources of evidence | 9 | A total of 1,658 records were identified across four databases. After removing 384 duplicates, 1,274 records were screened, and 1,091 were excluded based on titles and abstracts. Full texts of 183 articles were assessed, with 105 excluded for reasons such as inactive participants, absence of RT, or missing SmO₂ data. In total, 78 studies met the inclusion criteria and were included in the scoping review. | Results (General characteristics of selected studies) |
| Characteristics of sources of evidence | 10 | Data were charted for all 78 included studies, capturing key characteristics such as study design, participant demographics (age, sex, training status), sample size, assessed muscles, NIRS device type (CW, FD, or TD), exercise modalities (isometric, concentric, eccentric, isokinetic), training loads (%1RM or %MVC), external interventions (e.g., BFR, hypoxia, supplementation), and primary NIRS-derived outcomes (SmO₂, tHb, HHb, O₂Hb). | Results (Topics addressed by the studies) |
| Results of individual sources of evidence | 11 | Table 3 summarizes the key characteristics of all 78 studies included in this review. It documents participant demographics, experimental methods (NIRS devices, muscles examined, exercise types), and measured outcomes. | Results  (Supplementary materials) |
| **DISCUSSION** | | | |
| Summary of evidence | 12 | This scoping review mapped the current use of NIRS to monitor muscle oxygenation during resistance training. NIRS shows promise as a non-invasive tool for assessing acute physiological responses, including SmO₂ and THb, which may help clarify fatigue and internal load. However, more research is needed to determine its effectiveness for tracking metabolic stress and long-term adaptations. The 78 included studies were heterogeneous but generally fell into four themes: physiological responses during contractions, individual differences, training method comparisons, and external interventions. | Discussion |
| Limitations | 13 | The included studies showed substantial heterogeneity in their objectives, muscles examined, NIRS devices used, participant characteristics, and resistance-training protocols, which limits the ability to compare findings directly across studies. As a scoping review, the study did not include a formal risk-of-bias assessment, which restricts conclusions about the methodological quality of the included evidence. Variability in coaching methods, athlete profiles, and measurement approaches further reduces the ability to identify consistent patterns or draw strong inferences. | Discussion (Limitations) |
| Conclusions | 14 | NIRS use in resistance training is still emerging. Although it provides a non-invasive method to monitor internal load, current evidence does not support its ability to predict hypertrophy or strength outcomes. The relevance of NIRS-derived variables remains unclear, highlighting the need for rigorous studies to determine how it can inform practice. | Discussion (Conclusions) |
| **FUNDING** | | | |
| Funding | 15 | This work was supported by the Canadian Space Agency [grant number: 21FAQAMA07]. | Funding Section |

**Appendix S1**: PRISMA-ScR Checklist
